# Historical Sketch of the Mad River Dental Society

**Published:** 1883-04

**Authors:** George Watt


### Historical Sketch of the Mad River Valley Dental Society.

BY GEORGE WATT.

[Read in skeleton at a meeting for the reorganization of the society at Dayton, Ohio, October 24, 1882, and published by request of the society.]

The writer makes free to state, in justice to himself and the society, that the book containing the constitution, by-laws and minutes of the society was not accessible, and consequently he had to depend almost exclusively on his memory, therefore, this sketch is not what it ought to be.

In the early autumn of 1859, Dr. A. A. Blount came to Xenia and, with Dr. George L. Paine, called at the office of the writer, and after some miscellaneous conversation, the formation of a local society was spoken of, and before parting we set a time for a preliminary meeting in Springfield. In the absence of the minutes I cannot positively fix the date of this informal meeting, but if my memory is not at fault, a valuable paper sent by Dr. W. A. Pease was read. It was entitled, "Thoughts for Dentists," and may be found in volume xiii. of the DENTAL REGISTER, page 277.

The first regular meeting was held in Springfield, November 17, 1859. I can recall the presence of Drs. M. M. Oldham, Ramsey and Blount, of Springfield; Drs. G. L. Paine and G. Watt, of Xenia; and Dr. J. H. Paine, of Franklin, now of Middletown. At this meeting Dr. G. L. Paine read an "Essay on Crown Cavities in Molar Teeth," which may be found in the DENTAL REGISTER, in volume xiii. at page 297, and probably at the same meeting Dr. M. M. Oldham read a paper on "Impressions."

On the first Tuesday of January, 1860, the society met at Xenia. Dr. Taft's editorial notice says it was composed of dentists from Bellefontaine, West Liberty, Urbana, Springfield, Dayton and Xenia. A paper was read at this meeting by Dr. J. H. Paine on "Preparation and Filling a Simple Cavity in the Right Superior Cuspid, Posterior Proximate Surface." Dr J. Taft and some others united with the society, I think, at this meeting. A code of ethics and a fee bill were adopted.

The next meeting was in Urbana, the first Thursday of April, 1860, this being the fourth quarterly meeting of this society. This meeting was held at the office of Dr. B. A. Rose. By this meet ing and its doings I am reminded that Dr. G. Watt was the first President, and Dr. Geo. L. Paine the first Secretary of the society. At this meeting Drs. Pease and Bradley, of Dayton, and Drs. Harris and Williams, of West Liberty, were elected members. There was a discussion on mechanical dentistry during the day, and at the evening session Dr. Clippenger read a paper on "Taking Impressions in Plaster-of-Paris." This paper may be found in volume xiii. at page 628 of the DENTAL REGISTER.

But for a few meetings following, the recollection is too indistinct to rely on, and no minute account of these meetings can be given in the absence of the minutes.

At one meeting Dr. Blount read a paper on "Fang filling," and Dr. Rose one on "Treatment of Alveolar Abscess." The first is found in volume xiv. of the DENTAL REGISTER, at page 70; and the second is found at page 81 of the same volume. At this meeting the society adjourned to meet in Bellefontaine, the first Thursday of October, at the office of Dr. Clippenger, at 2 P.M.; and Drs. Palmer, Bradley and Watt were appointed essayists. In accordance with this appointment, Watt read a paper entitled "Thoughts on Caries," which may be found at page 270 of volume xiv. of the DENTAL REGISTER, and is also included in "Watt's Chemical Essays," and is, in substance, used as an appendix to "Taft's Operative Dentistry," thus giving it a much broader circulation than its author or the society anticipated.

But without minutes or memorandum, it is found impracticable to do anything like justice to the noble, energetic little society. An interesting item for the journals could be made by preparing a summary of the work done by it—the number of pages furnished the periodicals, and the general character of the papers read before it. If no one else can be induced to take up this good work I hope to be able to do something in this direction ere long, but would much prefer that an able pen, and one with more leisure should make the record. If this were done, I verily believe all would be surprised to learn how much work it has performed, and would be convinced that our worthy member, Dr. Berry, did not overstate the fact when he said this has been the best of our local societies.

The late civil war was hard on our little society, taking many of its most active members; and as it had been so liberal and had manifested so much of the missionary spirit as to take in members of the profession somewhat objectionable, hoping thereby to elevate and improve them, it was found impracticable, with the reduced membership, to manage them. Rather than see the beloved and endeared little society take a lower stand in ethics, a few members, the very best friends of the society, met *exactly on time*, and dissolved it by a *sine die* adjournment, literally putting the good little society to death to save its life, or rather to save its honor. But it may be said in pitiful pun that the *sine die* was not a sign it died ; for, in a short time a new society with the same name and substantially the same constitution and by-laws was organized, the objectionable members being left out.

The new society prospered for a time, but for reasons not calling for discussion here and now, there has been a suspension of the meetings for some years. But now, mainly, I believe, through the indomitable energy of Dr. Berry, it is proposed to revive it; and here we are. Let us resolve, here and now, each and all, that the Mad River Valley Society shall renew the vigor of its past life, and display the energy and activity of its early years.

I have found it impossible to do justice to the subject assigned me, as I have had to rely mainly on a treacherous memory.

Though a little foreign to the duty imposed, perhaps, may I not allude to the fact that probably no other dental society of its size includes two dental journals, one the oldest now in existence, and the other almost the newest.

The Society ought to find no difficulty in having its transactions published. I feel sure it will find ready help in laying its thoughts and actions before the profession. And while it is not for me to suggest, I would say, nevertheless, that it would probably be a good plan to regard papers read before the Society as referred to both journals; but, of course, the Society must do as it thinks best in this, as in other things. It is under no constraint to give its papers to either of them; but I doubt not the REGISTER, and I know the *Journal* will be pleased to lay before the profession any essay or paper which the Society regards as worthy of publication.

Before closing, it may be well to allude to some miscellaneous events, without much regard to date, which may give additional light as to the character of the Society.

A member was reported by the committee as worthy of a reprimand before the Sociaty for unprofessional conduct. Dr. J. Taft was appointed to discharge the disagreeable, but apparently necessary duty. While the words and tone were mild and sympathetic, the reprimand, taken as a whole, was most fearfully severe, and in all the experience of the writer, so serious an impressian was never before made and felt in any society. Not a member present failed to be most seriously impressed by this unique transaction. It is not necessary now to give name and date. The meeting at which this occurred was held in June, and at it Dr. Watt read a volunteer paper, written when bed-fast and in great agony—a paper with a history. Its title is "Professional Longings," and it was sent to the *Dental Cosmos* and rejected, and without reporting this fact, it was read, by way of appeal, word for word, as rejected, and the Society by unanimous vote requested it for publication. Then the fact of its rejection was told, and another vote requested, and it was given, with enthusiasm, unanimously again.*

I mentioned that the war was hard on the Society. I can not pretend, at this late day, to name those who went into the service of our country. Palmer, Blount, Jones, the two Paines, Watt, and many others. The heroic Palmer, in leading a charge at Gettysburgh, it is said, fell riddled with nine bullets. Jones was killed by accident at the burning of Pike's Opera House.

Some of our members, at least three, carried the banner of American dentistry into Europe, and to name Wright, Blount and Williams, is proof that the flag was not lowered.

Perhaps no other society of the size of ours has furnished so many teachers for our dental schools, or so many editors of dental periodicals. Besides, we have in our worthy President the first alumnus of the first dental college west of the Alleghenies, and the second in the world; and the laudable example set by him throughout his whole career, has done much to elevate and keep erect the tone of professional etiquette. It would take a bold, reckless alumnus to do a mean or low thing in his presence.

Nor has the little Society ever been niggardly. When it was proposed to make Dr. C. S. Barnum a pecuniary acknowledgment for his free gift to the profession, the Society, by a unanimous vote gave all the money in its treasury. This is but a type of its liberality. But what shall I say more? The time would fail me to tell how much the profession is indebted to this little Society for its progress—for its great attainments in combatting disease,

---

* In addressing a large meeting a few years ago, the writer made a quotation from this article, and a Baptist minister hinted that he was guilty of plagiarism, in not giving credit to Joseph Cook, and would not be convinced till shown the article as it first appeared in the DENTAL REGISTER.

and in replacing lost organs. A careful review will convince any of you that our worthy President was correct in claiming that this has been the best of our local societies.

---

MENDOTA, ILL.

EDITOR REGISTER.—A few days ago I saw a case of retarded eruption of the third molars, in the mouth of a lady in her *fifty-eighth* year. An upper one erupted about two years ago; the other three are in process of eruption now. The cusps can be plainly felt with the finger. At times there is considerable pain and general constitutional disturbance, seemingly due to this cause. I do not know how *rare* this is, but think it of sufficient interest to be made public. K. C. M.

